# Availability, Utilization, and Health Providers' Attitudes Towards Safe Abortion Services in Public Health Facilities of a District in West Bengal, India

**DOI:** 10.1089/whr.2019.0007

**Published:** 2020-03-06

**Authors:** Souvik Pyne, T.K.S. Ravindran

**Affiliations:** ^1^ The YP Foundation, Policy Engagement, New Delhi, India.; ^2^ Achutha Menon Centre for Health Science Studies, Thiruvananthapuram, India.; ^3^ International Institute for Global Health, United Nations University, Kuala Lumpur, Malaysia.; ^4^ School of Public Health, University of the Witwatersrand, Johannesburg, South Africa.

**Keywords:** abortion services, health providers', attitudes, health system

## Abstract

***Background:*** The provision of safe abortion services upholds the realization of justice in sexual and reproductive health. Many state-level studies in India have identified poor availability of abortion services in the public sector and negative attitudes toward abortion among health providers, as potential barriers to access.

***Materials and Methods:*** A cross-sectional study was done to document the availability and utilization of medical termination of pregnancy (MTP or abortion) services and to assess public sector health providers' attitudes towards safe abortion. It was carried out in a representative district of West Bengal, using a facility checklist and a validated attitude scale.

***Results:*** Only 11 of 42 public health facilities had both trained doctors and equipment to provide MTP services. Twelve facilities provided MTP services, of which only three urban-based secondary-level facilities provided second trimester MTPs. There were female providers in just 2 of the 12 MTP-providing facilities. Among the 64 health providers interviewed, 40% were trained to provide MTP. According to the attitude scale, 38% had a negative attitude toward the provision of safe abortion services. There was no statistically significant association between attitudes of health providers and provision of MTP. However, there appeared to be a subtle process of gatekeeping in operation, such as making MTP conditional on acceptance of contraception, requiring the husband's consent, and so on.

***Conclusions:*** The study shows the poor availability of abortion services in public sector facilities in a district of West Bengal, although all public health facilities from the primary health center level upwards are authorized to provide abortion services.

## Introduction

Safe abortion to terminate an unwanted pregnancy is an important reproductive health need across the reproductive span for women of all educational levels, racial and ethnic groups, social and economic classes, and religions. The need to make safe abortion services available has been upheld in many international platforms and intergovernmental agreements on sexual and reproductive health and rights, and women's rights, among others.

The 2008 estimates of the World Health Organization show that unsafe abortions account for 13% of all maternal deaths in the world. According to a report published by the Guttmacher Institute in 2012, there are ∼222 million women globally who do not want to get pregnant but are not able to do anything about it.^1,2^ About 43.8 million abortions take place each year, and 21.6 million are unsafe.^†^^,2^ Eighty-six percent of all abortions occur in developing countries, which constitutes 98% of all unsafe abortions across the world.^4^ About 8 million women annually suffer from unsafe abortion-related complications requiring medical care; 3 million of them do not get the required care and 47,000 women die due to unsafe abortions each year.^1,2^

Target 3.7 of the UN Sustainable Development Goals (SDGs) focuses on ensuring universal access to sexual and reproductive health (SRH) services. Availability of and access to safe abortion services are integral components of SRH services and needed in order to fulfill the SDG promise of “leaving no one behind”.

According to 2015 estimates, 15.6 million abortions took place in India and only 22% of them were obtained from health facilities.^5^ Earlier estimates indicate that there are ∼4,600 deaths from unsafe abortion each year, which translates to ∼13 women dying per day and hundreds suffering complications.^6^

The provision of safe abortion services is one of the strategies to reduce maternal mortality and morbidity in India under the Reproductive and Child Health Programme, Phase II (RCH-II) in 2005.^7^ However, in 2010, there was only one health facility providing abortion services per 100,000 population, much lower than the current recommendation of one abortion center per 20,000 population.^8^ Various state-specific studies in Bihar, Jharkhand, Chhattisgarh, Madhya Pradesh, Uttarakhand, and Meghalaya and multistate studies such as the Abortion Assessment Project-India (AAPI) also found low availability of abortion services.^9–15^ There was not much improvement in the availability of abortion services for more than a decade spanning pre-2000 to 2011. In the National List of Essential Medicine 2015, both mifepristone and misoprostol are listed under “tertiary” category, making their availability at lower public facilities uncertain.^16^

Health providers' attitude is a potential barrier to accessing abortion services. This is especially true in India, where although abortion is legal for many indications, the health provider is vested with the authority to decide whether or not a woman is eligible for abortion according to the law. A study in the 1990s in Uttar Pradesh found that health providers often considered abortion as “not right” and as a means to achieve sterilization “targets.”^17^ According to the AAPI study, health providers in many states lacked the willingness to provide unconditional abortion services to women coming alone or unmarried or widowed/divorced women, whereas in Kerala, the husband's consent was sought.^15^ Similar perceptions were found among medical students done a study in Maharashtra.^18^ Various studies done among health providers in Chhattisgarh, Meghalaya, Uttarakhand, and Kerala showed that a high proportion harbored a negative attitude.^11,13,14,19^ Moreover, inaccurate or inadequate information on the part of providers especially with respect to newer techniques such as medical abortion constitutes a significant challenge to abortion access in India.

West Bengal is the fourth most populous state of India and accounts for 10% of overall registered abortions. It is not represented in any situational analysis on safe abortion services post-2000.^8^ This study sought to address this research gap.

## Methodology

### Objectives and design

This study aimed to assess the availability of safe abortion services in public health facilities, utilization of the same where abortion services are available, and examine health providers' attitudes towards safe abortion. It was a cross-sectional study that consisted of a facility survey and a questionnaire-based survey of health providers.

### Setting

The study was conducted in public health facilities of South 24 Parganas district, West Bengal, during June to August 2015. South 24 Parganas was a representative district of the state with respect to abortion ratio (abortions per 1,000 live births) and percentage (abortions per 100 known pregnancies). The figures of the district were 37 and 3.5, respectively, which were closest to the state's figures of 39 and 3.8, respectively.^20^

### Sample size and sample selection procedures

For the facility survey, we used multistage cluster sampling. Public health facilities under the Directorate of Health Services, Department of Health and Family Welfare, Government of West Bengal in South 24 Parganas district were stratified into urban and rural settings. In the urban setting, both district hospitals and one (out of four) state general hospital that had the highest bed strength were selected. So, in the urban setting, three facilities were selected. In the rural setting, two (out of five) subdivisions, viz. Sadar (Alipore) and Diamond Harbour, were randomly selected by throwing a die. All the public health facilities till the level of primary health centers (PHCs) under each subdivision were selected. Subcenters were excluded since they are not mandated to provide abortion services. Sadar (Alipore) subdivision had three rural hospitals (RHs), three block primary health centers (BPHCs), and seven PHCs. Diamond Harbour subdivision had 6 RHs, 3 BPHCs, and 17 PHCs. So, in the rural setting, 39 facilities were selected. Therefore, the total sample size was 44 facilities.

For the health providers' survey, a total of 64 doctors were interviewed, who were present at the health facility during the time of the researcher's visit. From the urban setting, three most experienced and qualified doctors available were selected from each facility. In the rural setting, all doctors available in each facility were selected.

### Data collection techniques

A facility survey tool including an observation checklist was used to record the availability of abortion services and utilization of each facility. District data extraction tool was used to record the availability and utilization from records maintained at the district level based on reporting from approved Medical Termination of Pregnancy (MTP) facilities.

The Achutha Menon Centre for Health Science Studies abortion scale^19^ tool was used to assess the health providers' attitudes towards safe abortion. The tool had the following sections. Background information of the respondent including training history and practice (if applicable) and an attitude scale (20-item Likert-type scale). Since the study focuses on MTP services, only the 16 case scenarios relating to MTP were used. Each question has five responses that are scored from 5 to 1. The average score of the 16 responses was used to determine the attitude—positive or negative. A cutoff of 3.800 was determined using the Receiver Operating Characteristic (ROC) curve by taking into account responses only for the 16 case scenarios relating to MTP service provision. A score of 3.800 and above was classified as positive attitude, whereas a score of <3.800 was classified as a negative attitude.

### Data analysis

Data entry was done using Microsoft Excel 2007 software and analysis was done using licensed SPSS (version 21) software. Frequencies and mean were used for descriptive data. Pearson's chi-square test, Fisher's exact test, and Mann–Whitney *U* test were done to check for associations.

### Ethical considerations

The interviews were conducted in an environment where the respondent felt secure and comfortable, ensuring desired privacy. The information given by the respondents and the identity of the respondents as well as their institutions were kept anonymous to ensure confidentiality. Written informed consent was obtained from each respondent before the start of the survey or interview.

Ethical clearance was obtained from the Institutional Ethics Committee of Sree Chitra Tirunal Institute of Medical Sciences and Technology, Thiruvananthapuram, India.

## Results

### Availability and Utilization of MTP Services

#### General characteristics of the facilities

The sample included 42 facilities in all. Appendix Table A1 depicts the general characteristics of the facilities regarding access, staffing pattern, necessary infrastructure, drugs, and supplies.

#### Facilities equipped to provide MTP services, availability, and service provision

Since the study focuses on MTP, we looked into whether facilities were equipped with trained staff, adequate infrastructure and drugs, and supplies to provide surgical or medical abortion services. We then examined the number of facilities that actually provided the services.

Of the 42 health facilities surveyed, 12 provided MTP services. Eight facilities provided both surgical and medical methods, one provided surgical abortion alone, and three facilities provided only medical abortion. Nonavailability was often the result of a mismatch between availability of equipment and of trained professionals, especially at the secondary and primary care level.

The higher level facilities and six of the nine RHs had a trained doctor as well as full equipment, and all of them provided abortion services at the time of the study. Out of the three nonproviding RHs, one had neither a trained doctor nor equipment, another had only a trained doctor but not the full set of equipment, and another was fully equipped but lacked a trained doctor.

None of the nine BPHCs had both a trained doctor and equipment. One had a trained doctor but lacked equipment for surgical abortion, and so it provided Medical Methods of Abortion (MMA) exclusively.

Only 2 of the 24 PHCs provided MTP services. Two PHCs had both trained doctors and equipment, but only one provided both medical and surgical methods of abortions, whereas the other had discontinued both methods some time ago. Four PHCs had trained doctors but were not fully equipped, and none of them provided even MMA. Eighteen PHCs had neither a trained doctor nor was fully equipped, but one PHC provided MMA.

We visited the 12 facilities that provided MTP services and looked into characteristics of MTP service provision, some of which are also depicted in Table 1. Although the MTP Act permits termination of pregnancy up to 20 weeks, only the three higher level facilities located in urban areas provided second trimester abortion, whereas MTP up to 12 weeks was available in 6 of 12 MTP-providing facilities. The remaining three facilities provided only medical methods of abortion—two up to 8 weeks and one up to 6 weeks of gestation.

**Table 1.** Availability and Aspects of Medical Termination of Pregnancy Service Provision

MTP service equippedness and providing status (N = 42)	n (%)
Whether equipped to provide MTP services^a^	
Yes	11 (26.2)
No	31 (73.8)
Whether MTP services are provided^b^	
Yes	12 (28.6)
No	30 (71.4)

Distribution of MTP availability across facility types (N = 42)					
	Having trained doctor and equipped				Total
	Yes		No		
	Whether provided?				
	Yes	No	Yes	No	
District hospital	2	0	0	0	2
State general hospital	1	0	0	0	1
Rural hospital	6	0	0	3	9
Block primary health center	0	0	1	5	6
Primary health center	1	1	1	21	24

Distribution of MTP provision across facility types (N = 42)					
	Whether providing MTP				Total
	Yes			No	
	Surgical only	Medical only	Both		
District hospital	0	0	2	0	2
State general hospital	0	0	1	0	1
Rural hospital	1	1	4	3	9
Block primary health center	0	1	0	5	6
Primary health center	0	1	1	22	24

Characteristics of MTP-providing facilities (N = 12)	n (%)
Signboard displaying MTP service availability	6 (50.0)
Gestation age	
Up to 6 weeks	1 (8.3)
Up to 8 weeks	2 (16.7)
Up to 12 weeks	6 (50.0)
Up to 20 weeks	3 (25.0)

^a^ Equipped means to having both trained doctor and equipment for MTP.

^b^ Provided means was delivering MTP services (either of medical and surgical or both).

MTP, medical termination of pregnancy.

#### Utilization

Owing to poor and nonuniform record keeping, the quality of utilization data across all the MTP-providing facilities may underestimate the number of MTPs carried out. Since MMA records were missing in many facilities, we decided to consider only surgical MTP cases for looking into the utilization patterns. These are presented in Table 2. The total number of surgical procedures carried out in nine facilities in 3 months was incredibly low, roughly an average of 1.5 procedures per facility per month, with >80% being performed in the higher level facilities. The proportion of MTPs using manual vacuum aspiration method was much higher in lower level than in higher level facilities. There were 6 (of 12) facilities where both the MTP records and records of the evacuation of incomplete abortion cases were maintained, which we examined. These showed a many fold higher number of evacuations of incomplete abortions as compared with induced cases; cumulatively the number of incomplete abortions (207) was ∼10 times higher than the number of women seeking induced abortions (21).

**Table 2.** Utilization Patterns for Surgical Methods

Dimensions of utilization	Higher facilities (total = 33)	Lower facilities (total = 7)	Grand total (40)
Methods used			
MVA	3	5	8
Other surgical	30	2	32
Gestation age			
Up to 8 weeks	20	3	23
9–12 weeks	7	2	9
13–20 weeks	3	0	3
Not known	3	2	5
Age of abortion client (in completed years)			
<19	2	0	2
20–29	13	1	14
30–39	16	4	20
≥40	1	0	1
Not known	0	2	2
Recorded reasons for termination			
Danger to life	1	0	1
Failure of contraception	3	3	6
Socioeconomic	0	1	1
Wants ligation	0	1	1
Not known	29	2	31

Postabortion contraception provided	(Total = 29)	(Total = 6)	(Grand total = 35)
Condoms	0	0	0
Oral pills	5	3	8
IUDs	3	0	3
Sterilization	21	3	24

Higher facilities mean district hospitals and state general hospitals. Lower facilities mean rural hospitals, block primary health centers, and primary health centers.

IUDs, Intra-Uterine contraceptive Device; MVA, manual vacuum aspiration.

Most women undergoing abortions were in the age group of 30–39 years. Most of the MTPs took place within 8 weeks of gestation. Second trimester MTPs took place only in higher level facilities. Since most data were not from MTP registers and instead from operation theater registers, the recorded reasons for termination could not be traced in the majority of the cases. Postabortion contraception provided was heavily skewed toward sterilization.

### Health Providers' Attitudes

#### Characteristics of health providers

A total of 64 doctors were finally interviewed. In this section, the term “health providers” refers only to doctors in public facilities.

A majority (78.1%) of the health providers were male. Their age ranged from 27 to 70 years with the mean age of ∼43 years. Hindus were the majority (90.6%), and most (89%) of the health providers were married. Most (71.9%) of the health providers had MBBS degrees. Twenty-six of the health providers (40.6%) were trained to perform MTPs.

#### Attitudes towards safe abortion

Attitude score of each provider was calculated based on their responses to a 5-point Likert-type scale having 16 statements. These were 16 specific vignettes of women seeking abortion and asked whether they would provide abortion in that specific circumstance. Their responses ranged from “Never” to “Always.” One of the health providers failed to give responses to several statements, who was excluded from the analysis of data. The cutoff value for positive and negative attitudes on a scale varying from 1 to 5 was 3.800. The mean attitude score was calculated to be 3.880, which was more than this cutoff. Almost 62% of the health providers were found to have a positive attitude towards safe abortion. Mean attitude score distribution of health providers with positive and negative attitudes was calculated for subsets of those from MTP services providing facilities, those from MTP-equipped facilities (having equipment and trained doctor), and those who were MTP trained. All of these are depicted in Table 3.

**Table 3.** Aspects of Health Providers' Attitudes

Attributes	Mean ± SD/n (%)
All health providers (*N* = 63)	
Attitude mean score	3.880 ± 0.478^a^
Attitude towards safe abortion	
Positive	39 (61.9)
Negative	24 (38.1)
Health providers from the 12 facilities providing MTP services (*N* = 30)	
Attitude mean score	3.860 ± 0.482^a^
Attitude towards safe abortion	
Positive	18 (60.0)
Negative	12 (40.0)
Health providers from the 11 facilities equipped to provide MTP services (*N* = 28)	
Attitude mean score	3.839 ± 0.466^a^
Attitude towards safe abortion	
Positive	17 (61.7)
Negative	11 (39.3)
MTP-trained health providers (*N* = 26)	
Attitude mean score	3.793 ± 0.442^a^
Attitude towards safe abortion	
Positive	16 (61.5)
Negative	10 (38.5)

^a^ Highest possible score is 5 and lowest possible score is 1.

SD, standard deviation.

The mean attitude score of each of the statements in the attitude scale was individually calculated to look for trends across them. Appendix Table A2 depicts them. The highest mean scores were for “fetal anomaly” followed by “rape” and “trisomy 18.” The lowest mean scores were for “cleft lip in a primiparous woman” and “mental health being affected in a married primiparous woman.” The patterns depict that providers seemed to be more willing to provide abortion services for reasons of social stigma (pregnancy outside marital relationship), severe degrees of fetal anomaly than for contraceptive failure, first pregnancy within marriage, and mental health concerns of the client.

#### Factors influencing attitudes towards safe abortion

The associations between attitudes of health providers towards safe abortion with their sociodemographic characteristics and professional characteristics were examined by chi-square tests (when any cell value was <5, Fisher's exact test was done). None of the variables came out to be statistically significant.

Mean attitude scores of health providers segregated by their different sociodemographic and professional factors were calculated and tested using Mann–Whitney *U* test. But no statistical significance was found. Thus, we decided to compare mean attitude scores against the cut-off value and found the following. The religion of the health provider made a difference: the mean score of Hindus was above the cutoff (*i.e.*, positive), whereas that of other religions was below it (*i.e.*, negative). Similarly, overall training status too made a difference, with MBBS-trained providers' mean score being above the cutoff (*i.e.*, positive) and those with postgraduate specialization below it (*i.e.*, negative). Also, the mean score of health providers in higher facilities was lower than the cutoff (*i.e.*, negative), whereas those in lower facilities above it (*i.e.*, positive).

We found no association between attitudes towards safe abortion and abortion provisioning by health providers. However, there seem to be subtle ways in which subversion of the services happened. Instead of an outright denial to service provision, there were various forms of gatekeeping to restrict women's access to abortion services through strategies such as provision conditional on acceptance of contraceptive methods, requiring husbands' consent, refusal to unmarried clients, dissuading primigravida women, reluctance to provide in the public facility, and channelizing to their private chambers.

## Discussion

### Availability

This study found that the availability of abortion services in the public sector was poor. Considering the total population of the randomly selected subdivisions according to census 2011 data, the availability translates into 1 abortion providing facility per 250,000 population or 0.4 facilities per 100,000 population. This is a much lower availability than the Federation of Obstetric and Gynaecological Societies of India recommendation (1 per 20,000 population) and AAPI study findings (4 per 100,000 population), the latter done more than a decade ago.^15^ The low availability was despite the proximity to Kolkata metropolitan city of one of the randomly selected subdivisions in our sample and another subdivision including a major urban area. The two district hospitals of the district were in these subdivisions, and the other subdivisions have relatively poorer health infrastructure. Thus, the actual public sector availability in the whole district is worse than what this study found. Even if the higher level facilities had availability of MTP services, access to them was difficult for most women owing to factors such as long distances and associated indirect costs, multiple appointments, multiple contact points, and long waiting times. The study also found that there were female providers in just 2 of all the 12 MTP-providing facilities, which could also have been a deterrent to women seeking MTP services. Such lack of female providers was reported in the AAPI study of more than a decade ago.^15^

The formal private sector in the study area had just one MTP-licensed facility in the whole district. Given such scarcity of authorized MTP service delivery facilities, the unlicensed private sector, as well as the unqualified informal sector, had captured the “market.” They were also reported to have fewer formalities, round-the-clock and prompt services, proximity to women's home, greater perceived privacy owing to less crowd, and fewer staff members. Our observations in the field hinted that there might be mushrooming of many small nursing homes, most of which lacked basic infection-control standards. The unqualified informal sector presumably may have even lower standards of service provision. Women in dire need of services are bound to choose such unsafe settings, because better equipped private facilities are few, and financially unaffordable.

Medical abortion drugs were freely available in the study area: in chemist shops, with informal providers, and even in grocery shops. According to many respondents, these were considered as the first line of action to terminate unwanted pregnancy irrespective of gestational age. These findings were similar to a study on medical abortion in Bihar and Jharkhand, but unlike nonprescription sales being limited there, it was apparently more easily available here.^21^

### Utilization

In whatsoever public facilities abortion services were being provided, the utilization data showed meager numbers of only 40 abortions for 3 months, and 33 of them had been carried out in the two urban-based district hospitals and the state general hospital. These low numbers do not at all depict that the overall abortion cases are few, it is just that the public sector is not the popular choice. Although we did not have private sector utilization data, it can be safely presumed that it follows a similar trend of skewed private sector utilization as reported in a six-state AAPI study (87%) and MTP situational analyses in Bihar (85%) and Jharkhand (88%).^9,10,15^

Second trimester abortions being restricted to urban-based higher facilities are similar to findings from the AAPI study and other state-level studies done in Bihar, Meghalaya, Chhattisgarh, and Uttarakhand.^9,11,13–15^

There was an incidental finding of very high number of incomplete abortion cases being reported in facility records. One possible explanation is that women are mostly self-inducing abortions using medical abortion pills or taking them from informal providers and later presenting to the government facilities as incomplete abortion cases. These get misreported as spontaneous abortions, especially from the urban-based higher level facilities that received a huge number of incomplete cases. This depicts that most abortions take place outside health facilities and the national estimation of abortion incidence study suggests the same.^5^

### Attitudes towards safe abortion

Health providers' attitudes towards safe abortion was assessed using a validated scale that was used among obstetrics and gynecology professionals in Kerala. The Kerala study found that only 40% of health providers had a positive attitude and it was more likely among Hindus than among other religions, public sector doctors than private sector doctors, and women than men. In our study, the attitude of a majority of health providers (62%) was positive. There was no association between MTP provision and the attitude of providers in the subsample of MTP-trained doctors. However, MTP services were being provided wherever a facility was both equipped and had an MTP-trained doctor. The sample characteristics varied between the studies. Although our study had only public sector doctors, the Kerala study had predominantly private sector doctors; although our study had only two women providers in the MTP-trained subsample, the Kerala study had a majority of women providers. Although our study had only Hindus in the subsample, the other had other religions too.^19^ These differences may have contributed to the absence of an association between provider attitude and abortion provision in our study. Unlike in Kerala, where there is a strong association between the attitude of predominantly private sector women doctors and service provision, among the male public sector providers in West Bengal, attitude is not a big factor in influencing MTP service providing status. However, there were various forms of gatekeeping and subtle ways of denial of abortion services.

### Limitations of the study

The major limitation of the study was the exclusion of the private sector. Although there was only one licensed formal private facility in the district, service provisioning by the private sector was reported to be in abundance. There was poor record keeping by the facilities and at the district level, which was a limitation to a complete understanding of service provision and utilization patterns.

## Conclusions

The study attempted to find out the abortion services scenario in a representative district of West Bengal. It highlighted the limited availability of safe abortion services in the public sector, and various factors that affect its utilization adversely. All these contributed to the creation of constraints from the public facilities in the provisioning of safe abortion services. This gap had been promptly filled up by the unlicensed formal and informal private providers, whose service provisioning has concerns around medical safety, affordability and legality.

It is perturbing that even after more than four decades of the legalization of abortion in the country, women in a state like West Bengal have to depend on and seek unsafe services from unlicensed private and informal providers. There is scant scientific literature on abortion from West Bengal, and this study is a modest contribution to the evidence base of the state. The study results resonated with many abortion-related studies conducted in different parts of the country and during different periods. This is harsh testimony to the fact that safe abortion services are neglected throughout the country and there has been hardly any improvement in availability for more than a decade.

The 2002 MTP Act amendment was meant to facilitate licensing of MTP provision by private health facilities, but the findings of this study suggest that the amendment has not had the desired effect at least in this part of the country. Although the RCH-II and Reproductive, Maternal, Newborn, Child, and Adolescent Health guidelines feature safe abortion services, there seems to be much deficiency in translating this aspect of the policies into action.

Viewing from a human rights perspective, access to safe abortion services comes under the ambit of the right to health care as upheld in Article 12 of International Covenant on Economic, Social, and Cultural Rights. The United Nation's Human Rights Council has established links between women's equality and the availability of reproductive services including abortion. Thus, reduced availability of medical service needed only by women also amounts to a violation of rights to nondiscrimination and equality. Therefore, it is the duty of the State to upscale efforts to implement comprehensive abortion care services in public sector facilities more efficiently. Availability of at least MMA at primary health care facilities level is essential, and MMA drugs (mifepristone and misoprostol) should be put under “all levels” category of essential drugs list. Moreover, there is a pressing need to sensitize the health providers on safe abortion from a rights and gender perspective rather than a medical service to be delivered at the whims of the health provider.

## Abbreviations Used

AAPI: Abortion Assessment Project-India
BPHCs: Block Primary Health Centers
MTP: Medical Termination of Pregnancy
PHCs: Primary Health Centers
RCH-II: Reproductive and Child Health Programme, Phase II
RHs: Rural Hospitals
SD: Standard Deviation
SDGs: Sustainable Development Goals
SRH: Sexual and Reproductive Health

## Notes

^†^ According to the WHO, unsafe abortion is a procedure for terminating an unwanted pregnancy either by persons lacking the necessary skills or in an environment lacking minimal medical standards, or both.^3^

## Appendix

**Appendix Table A1.** General Characteristics of the Health Facilities

Characteristics (N = 42)	*n* (%)
Type of facilities	
District hospitals	2
State general hospitals	1
Rural hospitals	9
Block primary health centers	6
Primary health centers	24
Distance from road accessible by public transport	
On road/<1 km	35 (83.3)
1 to <3 km	2 (4.8)
3 to <6 km	4 (9.5)
≥6 km	1 (2.4)
Transport facilities^a^	
Bus	25 (59.5)
Train	6 (14.3)
Boat	1 (2.4)
Auto/cycle rickshaw	38 (90.5)
Cycle van	9 (21.4)
Motor van	13 (31)
Trekker	6 (14.3)
Metro	1 (2.4)
No. of doctors	
0	3 (7.1)
1	18 (42.9)
2–5	15 (35.8)
6–9	3 (7.1)
≥10	3 (7.1)
No. of nursing staff	
1	15 (35.7)
2–5	10 (23.8)
6–9	10 (23.8)
≥10	7 (16.7)
Facility characteristics^a^	
Covered waiting area with seating	37 (88.1)
Drinking water for clients	37 (88.1)
Toilet for clients	37 (88.1)
Counseling room/area	19 (45.2)
Generator/inverter	22 (52.4)
Privacy in consultation room	21 (50.0)

^a^ Percentages would exceed 100% when added, categories being mutually not exclusive.

**Appendix Table A2.** Attitude Mean Scores of Statements in the Attitude Scale

Sl. No.	Statements	Mean ± SD^a^
1	A 20 weeks pregnant married woman, first time pregnant, whose mental health will be affected if the pregnancy is continued	2.984 ± 1.476
2	A 16 weeks pregnant married woman whose physical health will be affected if the pregnancy is continued	3.571 ± 1.103
3	A 16 weeks pregnant unmarried woman whose physical health will be affected if the pregnancy is continued	4.320 ± 1.029
4	A 20 weeks pregnant unmarried woman whose mental health will be affected if the pregnancy is continued	4.020 ± 1.326
5	A 16 weeks pregnant woman whose pregnancy is a result of rape	4.841 ± 0.410
6	A 19 weeks pregnant 17-year-old girl whose pregnancy is because of incest	4.540 ± 0.877
7	A 19 weeks pregnant married woman whose USG has shown evidence of a congenital disorder, trisomy 18	4.794 ± 0.481
8	A 20 weeks married woman who is pregnant because of failure of laparoscopic tubectomy she had undergone	3.841 ± 1.167
9	A 16 weeks pregnant woman whose pregnancy is the result of rape by husband	3.270 ± 1.247
10	A 20 weeks pregnant married woman whose mental health will be affected if the pregnancy is continued	3.540 ± 1.189
11	A 16 weeks pregnant married woman who is pregnant because of the failure of oral pills used inconsistently by her	3.794 ± 1.166
12	A 6 weeks pregnant married woman who is pregnant because of the failure of the condom used by her husband	3.794 ± 1.233
13	A 16 weeks pregnant married woman who is pregnant and an USG has showed evidence of fetal anomaly	4.857 ± 0.535
14	A 6 weeks pregnant married woman who is pregnant within 10 weeks of her husband undergone vasectomy	4.079 ± 1.126
15	A 16 weeks pregnant married woman who is pregnant when her husband has been abroad for a year	4.000 ± 1.231
16	A 16 weeks pregnant married woman who is pregnant for the first time and the USG shows evidence of a cleft lip	1.841 ± 1.181

^a^ Highest possible score is 5 and lowest possible score is 1.

SD, standard deviation; USG, Ultrasonography.
